# Comprehensive Model for Physical and Cognitive Frailty: Current Organization and Unmet Needs

**DOI:** 10.3389/fpsyg.2020.569629

**Published:** 2020-11-26

**Authors:** Fulvio Lauretani, Yari Longobucco, Francesca Ferrari Pellegrini, Aurelio Maria De Iorio, Chiara Fazio, Raffaele Federici, Elena Gallini, Umberto La Porta, Giulia Ravazzoni, Maria Federica Roberti, Marco Salvi, Irene Zucchini, Giovanna Pelà, Marcello Maggio

**Affiliations:** ^1^Geriatric Clinic Unit, Medical Geriatric Rehabilitative Department, University Hospital of Parma, Parma, Italy; ^2^Department of Medicine and Surgery, University of Parma, Parma, Italy

**Keywords:** aging, frailty, motoric cognitive syndrome, mild cognitive impairment, organizational models

## Abstract

Aging is characterized by the decline and deterioration of functional cells and results in a wide variety of molecular damages and reduced physical and mental capacity. The knowledge on aging process is important because life expectancy is expected to rise until 2050. Aging cannot be considered a homogeneous process and includes different trajectories characterized by states of fitness, frailty, and disability. Frailty is a dynamic condition put between a normal functional state and disability, with reduced capacity to cope with stressors. This geriatric syndrome affects physical, neuropsychological, and social domains and is driven by emotional and spiritual components. Sarcopenia is considered one of the determinants and the biological substrates of physical frailty. Physical and cognitive frailty are separately approached during daily clinical practice. The concept of motoric cognitive syndrome has partially changed this scenario, opening interesting windows toward future approaches. Thus, the purpose of this manuscript is to provide an excursus on current clinical practice, enforced by aneddoctical cases. The analysis of the current state of the art seems to support the urgent need of comprehensive organizational model incorporating physical and cognitive spheres in the same umbrella.

## Introduction

The term *aging* defines the changes occurring during an organisms’ life (da Costa et al., 2016). From a biological perspective, *aging* is associated with functional decline and cellular impairments resulting in a wide variety of molecular damage over time. All these changes affect physical and mental capacity (World Health Organization [WHO], 2018).

Aging population is the result of low immigration and reduced fertility (Christensen et al., 2009) with constant increased life expectancy (Ferrucci et al., 2008).

The rate of aging of the world population is increasing from 900 million in 2015, and the population older than 60 years is expected to reach 2 billion by 2050, mostly in low–middle socioeconomic level countries (World Health Organization [WHO], 2018). Nowadays, the number of people aged 80 and over is 125 million.

In the United States, the entire population will grow to 400 million people in the next 40 years. The 65-year age group and older will increase by almost two times, reaching 95 million people, 25% of the entire country population (Vespa et al., 2020).

Italy and Germany are the oldest European and World Countries. By 2030, almost 25% of the European population will be represented by seniors (Ferrucci et al., 2008).

The relationship between the older adults and the working age population, defined age dependency ratio, is used to define the level of support provided to the older population by the 15–64-year-old population (EUROSTAT, 2019).

In the next 5–10 years, the Italian population is expected to decrease, from 60.6 million in January 2017 to 54.1 million in 2065 (Istituto Nazionale di Statistica [ISTAT], 2018).

Furthermore, the geographic areas of longevity have been extensively studied. Many centenarians living in these “zones” are free of chronic diseases, completely independent in activities of daily living (ADL), and do no develop any condition of disability up to the age of 90 (Deiana et al., 1999; Ferrucci et al., 2008).

*Aging* cannot be considered a homogeneous process. When the intrinsic capacity, which is the sum of physical and mental capacities, is reduced or lost, a condition of frailty occurs (Cesari et al., 2006; Longobucco et al., 2019). Frailty, defined as a state of increased susceptibility to stressors (high or low temperature, acute illnesses, or injuries), implies the homeostatic dysregulation of many physiological systems (Fried et al., 2004). It may be characterized by low physical function, cognitive performance, or both, with increased difficulty or dependence in basic activities of daily life.

Frailty is a highly prevalent condition worldwide. For example, in a Malaysian over 60 institutionalized population, the prevalence of physical frailty and prefrailty was 56.6 and 40.7%, respectively (Murukesu et al., 2019). In the same country, in a community setting, the prevalence of cognitive frailty was 2.2%, while the prefrail persons were the 37.4% (Malek Rivan et al., 2019). The incidence of cognitive impairment was estimated in 7.1/100 persons per year (Rivan et al., 2020). A recent meta-analysis showed that the hazard ratio for the co-occurrence of both physical and cognitive frailty was 5.36 (Grande et al., 2019).

## Frailty as a Dynamic Process

In this manuscript, we underline the close relationship between the motor and cognitive components and their contribution to a predisability condition. We bridge the concepts of frailty and motoric cognitive risk syndrome, providing an operational interpretation (Verghese et al., 2019).

The progression of physical and cognitive frailty leads to physical disability and dementia. As suggested by some authors (Rossini et al., 2019), the evolution of mild cognitive impairment toward Alzheimer’s disease occurs in 50% of the patients.

At the same time, sarcopenia becomes a leading determinant of physical frailty and represents a reversible precursor of hypomobility or bed rest. These issues have been conceptualized in the operative definition of the Sarcopenia and Physical fRailty IN older people: multi-componenT Treatment strategies (SPRINTT) project, the most important randomized controlled trial on physical frailty (Marzetti et al., 2018).

If screening tests are combined to assess the physical and cognitive components of frailty (for instance, sarcopenia and mild cognitive impairment), the diagnostic accuracy of the prodromal of dementia is increased. In fact, the combined use of physical and cognitive frailty allows to detect the highest risk of developing dementia and disability (Grande et al., 2019).

Moreover, the widespread deposition of amyloid in the central nervous system of patients suffering from mild cognitive impairment and Alzheimer’s disease can contribute to the decline in physical and cognitive performances (Lauretani et al., 2020).

Therefore, our discussion regarding the pathophysiological mechanisms of frailty will be restricted to sarcopenia and cognitive frailty as determinants of frailty. We will also address the possible synthesis of these two conditions by discussing the motoric cognitive risk syndrome.

### History and Models

The term *frail older persons* was used for the first time by Bertha Adkins, past president of the Federal Council on the Aging, during a radio interview to describe “those people needing continuous social support due to accumulation of disabilities associated with aging.” Despite the increase in geriatric medicine over the last decades, a univocal definition of frailty is still missing (Pilotto et al., 2020).

Fried et al. (2004) identified frailty as a clinical syndrome of vulnerability with low functional supply and compromised capacity to face stressful conditions, resulting in multiple organ failure and adverse outcomes.

Rockwood and Mitnitski (2007) gave an alternative definition operationalizing frailty as a state of dysregulation of physiological systems estimated by functional state, multimorbidity, motoric and cognitive deficits, and social predisposing conditions, for outlining the risk of unfavorable events. All these predisposing conditions were enumerated into a preformed list called “Frailty Index” (Rockwood and Mitnitski, 2007).

Gobbens et al. (2010) moved toward a biopsychosocial model of frailty, a dynamic, multifactorial condition characterized by changes in one or more than psychological, social, and physical domains, and determining an increased risk of unfavorable outcomes.

### Definition

Despite the different approaches, most of the authors agree that *frailty* is a dynamic intermediate condition between a normal functional state and disability determining the decline of functional abilities (Walston et al., 2006).

*Frailty* has been also assimilated to a multidimensional geriatric syndrome featured by the decreased ability to recover homeostasis when a stressor event and the loss of functional reserves occur. Frailty affects physical, psychological, and social domains involving cognitive, emotional, and spiritual aspects (Longobucco et al., 2019).

Seventeen of the European elders show frailty. The increasing prevalence across European countries suggested the need of crossing the geriatric field and improving an appropriate diagnosis (Wleklik et al., 2020).

### Pathophysiology

The pathogenesis of frailty is multifactorial and includes age, acute and chronic diseases (multimorbidity), genetic heritage, loss of loved ones, and polypharmacy as risk factors (Gutiérrez-Valencia et al., 2018). Physical (inflammatory status, hormonal imbalance), psychological (stress and depression), and social factors are core determinants and components.

In a small frail cohort of elder patients, Leng et al. (2002, 2004a) found that lower hemoglobin and hematocrit levels inversely related with interleukin 6 levels and proxy of inflammatory status.

An increased activation of monocytes and macrophages has also been documented in frail patients (Leng et al., 2011; Ramanathan et al., 2013). Frailty was also linked to changes in hormonal milieu, namely low serum levels of insulin like growth factor-1 (IGF-1) and dehydroepiandrosterone sulfate (DHEAS) well-known anabolic hormones (Leng et al., 2004b; Puts et al., 2005; Shardell et al., 2009; Maggio et al., 2012, 2014).

Stress, depression, low activity levels, lower dietary protein, and micronutrient intake can accelerate the process of frailty (Fried et al., 1999). Other contributing causes of frailty (Strawbridge et al., 1998) include social isolation, alcohol abuse, smoking, chronic diseases, and polypharmacy.

A special contribution to physical frailty comes from sarcopenia and the decay of muscle quantity and quality, which can be considered its biological substrate (Xue, 2011; Clegg et al., 2013; Coelho et al., 2015; Morley, 2016).

According to the presence of multimorbidity, polypharmacy, sensory deficits, and loss of social support, we can distinguish prefrailty and frailty. Both forms are associated with increased risk of hospitalization and death (Newman et al., 2001).

Earlier recognition (catching signs and symptoms of physical and cognitive domains), diagnosis, and multimodal treatment are needed to prevent the progression of prefrailty into functional decline. This approach is also fundamental to attenuate the risk of morbidity, dependence, falls, mortality, social isolation, admission to care facility, and reduced quality of life (Longobucco et al., 2019).

Actually, there is no global evaluating scale available to address all the clinical aspects of this syndrome including sarcopenia, which is closely connected to physical frailty and requires a parallel evaluation (Cruz-Jentoft et al., 2019). We are still using many different physical, psychological, and social tools to explore different spheres of frailty in the context of comprehensive geriatric assessment.

#### Sarcopenia

Sarcopenia is a skeletal muscle disorder characterized by low muscle mass and quality. Nowadays, the most influential definition is presented by the “European Working Group on Sarcopenia in Older People” (EWGSOP), supported by the “Asian Working Group on Sarcopenia,” and updated as “EWGSOP2” in January 2019 (Cruz-Jentoft et al., 2019).

Sarcopenia is an age-related physical condition with a multifactorial etiology, including genetic and lifestyle factors and multimorbidity. The most important causes of sarcopenia are inactivity, eating habits, diseases, and medications.

Therefore, sarcopenic persons have a peculiar physical condition characterized by loss of muscle strength (quality) and mass (quantity). We can identify an acute (usually after surgery, during hospital admission, or in other conditions of immobility) and chronic sarcopenia especially due to prolonged inactivity and immobilization (Cruz-Jentoft et al., 2019).

Muscle aging is characterized by an imbalance between anabolic and catabolic pathways with reduced muscle proteins and myofibers (in particular type II fibers) frequently replaced by adipose tissue.

It is possible to diagnose sarcopenia combining different data, including motoric performance tests and measures of muscle mass and strength.

Given the related risks of functional decline, falls, frailty, and death, several studies are now focusing on easier and more accurate techniques to measure muscle mass.

In particular, the daily application of well-known techniques such as dual-energy X-ray absorptiometry (DEXA), magnetic resonance imaging (MRI), and bioimpedance analysis (BIA) is limited by the costs and complex analyses. In this scenario, B-mode muscle ultrasound is a promising technique for screening muscle mass and structure and in the future for diagnosing sarcopenia (Ticinesi et al., 2017).

Handgrip strength has been commonly used to measure muscle strength. EWGSOP2 suggests to identify cutoff gender dependent and explained by the different hormonal milieu (Maggio et al., 2013; Cruz-Jentoft et al., 2019).

Short Physical Performance Battery test (Guralnik et al., 2000), “Timed Up and Go,” and “Walking Speed Test” are the tests commonly used to assess the motoric performance and sarcopenia severity (Cruz-Jentoft et al., 2019).

Additional and useful information comes from SPRINTT Study. This trial was conducted with the double goal of finding a consensus on the identification of older adults with physical frailty and sarcopenia and to test the effectiveness of a multifactorial intervention in this specific population living in the community. A specific program of physical activity, dietary, and technological intervention was compared to a successful Aging Lifestyle Education program having as primary outcome changes in 400 m walking. The results of this trial are close to be published (Landi et al., 2017).

#### Cognitive Frailty

Cognitive frailty is an emerging concept and condition of reduced neuropsychological reserve where physical frailty and mild cognitive impairment (MCI) coexist. We are facing a heterogeneous geriatric condition where cognitive capacities are preserved or slightly reduced with preserved activities of daily living. Two MCI subtypes are potentially reversible cognitive frailty (physical frailty/MCI) and reversible cognitive frailty (physical frailty/pre-MCI subjective cognitive decline) (Panza et al., 2018).

Cognitive impairment is more frequently detected in physically frail patients. In this specific category, we can observe adverse clinical outcomes linked to physical (functional independence, hospitalization, and risk of death) and cognitive components of frailty [dementia, in particular Alzheimer’s disease (AD)]. Several studies are revealing the role of brain as the core not only for dementia but also for frailty syndrome. Physical activity has beneficial effects on the brain and muscle, suggesting that neuroprotection is a potential way to increase muscle function.

The research is also focusing on disease-modifying therapies targeting various forms of dementia and in particular Alzheimer’s type. Ongoing clinical trials (Murukesu et al., 2020) are testing feasible and promising treatments capable to slow down the natural course of the disease.

This is why growing attention should be payed to scenarios frequently occurring in clinical practice.

##### Scenario 1

Asymptomatic patients at high risk of dementia. This definition is presently applicable to overall healthy patients carrying genetic mutations that are pathogenic for AD or frontotemporal dementia (FTD) (guidelines for the detailed description of high-risk patients are fully described in SINDEM consensus paper by Bocchetta et al., 2016). The asymptomatic stage must be verified by the administration of questionnaires for cognitive symptoms followed by accurate neuropsychological and neurological examinations. The Clinical Dementia Rating (CDR) scale must be 0. In the case of familiar history of dementia, a genetic counseling and testing shall be performed together with an accurate analysis of the age of onset of symptoms and the timing for starting therapy. If the family carries pathogenic mutations, the use of biomarkers is considered useful just for follow-up but not for diagnostic purposes. If we consider this status, an early onset Alzheimer’s disease (EOAD) case (Stevens et al., 2011), in Italy, there are (2016) about 6,000 cases, 50% of which carry mutations of pathogenic genes.

##### Scenario 2

Patients with a prodromal stage of AD (IWG2) or mild cognitive impairment (MCI) prodromal to AD (NIA-AA). MCI is an intermediate stage between normal cognition and dementia, considered a clinical and neuropsychological condition typical of older persons’ brain.

The neuropsychological evidence of memory impairment is the main characteristic of this condition that does not fully meet the criteria of dementia.

Recent studies indicate that mnesic MCI (aMCI) precedes Alzheimer’s disease, with 50–60% of patients developing dementia and the remaining 50–40% stable in this condition or get back to normality.

Thus, it is fundamental to diagnose aMCI and to evaluate possibilities and timing of progression to dementia. The appropriate diagnosis will allow to plan organizational and rehabilitative interventions and to start therapies. The following criteria used to define MCI are based on cognitive questionnaires and screening tests [Mini-Mental State Exam (MMSE)], neuropsychological evaluation (including two tests for episodic memory, tests for language, visuospatial abilities, and behavioral scales with appropriate normative thresholds, functional scales, neurological examination, and CDR score of 0.5) (Cerami et al., 2017; Costa et al., 2017).

The capacity of identifying and diagnosing this condition in the first stages increases the probability of reducing health and social costs related to dementia.

Moreover, the ability to detect MCI can be harnessed in new clinical trials with potential disease-modifying experimental drugs. The combination of specific tests [i.e., hippocampal volumetric MRI, 18F-fluorodeoxyglucose (18F-FDG)-PET and lumbar puncture for cerebrospinal fluid (CSF) examination] is already helpful to identify MCI and predict its evolution into AD.

However, their widespread use in a large population is difficult given the high costs, low availability, and invasiveness. A meta-analysis conducted by an international consortium (Sachdev et al., 2013) has clarified the epidemiological features of MCI condition. Its prevalence in a population with 60 years and older is 5.9% and increases over time ranging from 4.5 to 7.1% in individuals of sixth and eighth decade, respectively. Based on such values, in 2016, around 735,000 Italians were estimated to be MCI clients. Another reason to target this type of patients (scenario 2) relies on the growing evidence that the prodromal stage seems the most responsive to experimental disease-modifying drugs (including those recently failed in the early/moderate AD stage).

##### Scenario 3

Patients with early AD condition defined by MMSE adjusted for age and education, score between 21 and 25/30, neuropsychological evaluation (including two tests for episodic memory, tests for language, visual–spatial abilities, and behavioral scales with appropriate normative thresholds, functional scales, neurological examination) and a CDR score of 1 (Cerami et al., 2017; Costa et al., 2017). In Italy, there are about 500,000 AD cases. Although it is not easy to count the participants in the early stage, using the CRONOS project, we can estimate that 60% of them—nearly 300,000 patients are in this stage (Vanacore et al., 2002).

Mild cognitive impairment and frailty require a multidomain approach including physical, nutritional, cognitive, and psychological one. It would be also important to start pharmacological and non-pharmacological treatments during the initial stages of cognitive frailty.

However, the need of standardized treatments is not supported by robust clinical trials.

### Interaction Between Physical and Cognitive Frailty: Motoric Cognitive Risk Syndrome

Cognitive impairment should be considered as an intermediate stage between “normal” aging and advanced dementia. It is also known that cognitive decline, known as cognitive frailty, coexists or even is preceded by conditions of physical frailty such as low mobility and gait impairment. Therefore, these preclinical conditions should be considered as a unicum (Montero-Odasso et al., 2012).

Verghese et al. (2012) validated the motoric cognitive risk (MCR) syndrome, the combination of initial cognitive decline (but without a diagnosis of dementia) and relevant functional impairment in older persons. The authors considered four diagnostic criteria: cognitive complaints assessed with the Consortium to Establish a Registry for Alzheimer’s (CERAD) questionnaire (Rossetti et al., 2010), slow gait speed, preserved activities of daily living, and absence of dementia. In this study, older participants meeting the MCR criteria had a global risk of developing dementia about three times higher, and risk of developing vascular dementia increased by about 12 times (Verghese et al., 2013).

Clinical extrapyramidal and other neurological signs such as tone or strength alone do not predict dementia (Waite et al., 2001).

None of the patients with only slow gait, and then without cognitive disorders, developed vascular dementia. These data support the need of a global patient evaluation including cognitive and physical dimensions (Verghese et al., 2013).

Furthermore, these authors investigated cognitive and risk factors profiles of five different subtypes of MCR and their respective risk of incident cognitive impairment: slow gait velocity MCR (MCRv), short stride length MCR (MCRsl), slow swing time MCR (MCRsw), high stride length variability MCR (MCRslv), and high swing time variability MCR (MCRswv). The MCRswv was associated with incident memory impairment, strengthening the role of MCRswv as preclinical marker of Alzheimer’s. One possible explanation is that oscillation time variability represents a higher level of gait control and proxy of cognitive function (Allali et al., 2016). Hippocampal regions, which oversee walking control, are damaged during early stages of Alzheimer’s disease (Fox and Schott, 2004). MCRsl was a predictor not only of cognitive decline but also of visual–spatial impairment, which is a typical clinical picture of Parkinson’s disease. These data are consistent with the notion that decreased stride length is the hallmark of synucleinopathies (Calabresi et al., 2006; Grabli et al., 2012; Allali et al., 2016).

Epidemiological population studies suggest that about 10% of the older persons are affected by MCR, and the presence of this syndrome represents a risk factor for disability (Verghese et al., 2014). Nowadays, MCR is detected with different tools and outcomes (Table 1) (Ferrucci et al., 2000; Jhoo et al., 2008; Callisaya et al., 2010; Herman et al., 2010; Montero-Odasso et al., 2011, 2018; Kowal et al., 2012; Meguro et al., 2012; Verghese et al., 2012; Bridenbaugh et al., 2013; Lord et al., 2013; Vannier-Nitenberg et al., 2013; Holtzer et al., 2014; Cruz-Jentoft and Sayer, 2019; Grande et al., 2020). Prevalence of MCR in Europe is around 8.0%, reaching 7.0% in the United States and 6.3% in Japan. It is estimated that the incidence is 65.2/1,000 inhabitants/year in people aged 60 or over (Maggio and Lauretani, 2019).

**TABLE 1:** Tests used in the main studies for the diagnosis of motoric cognitive risk syndrome and related clinical outcomes (modified from Verghese et al., 2014).

	**Study**	**Assessment Method**		**Outcomes**
		**Physical function**	**Cognitive complaint**	
**Cognitive Frailty**				
79	Italy (INTERCEPTOR Project), 2020		CDR = 0.5 (presence of mild cognitive impairment)	Conversion to Alzheimer’s disease: CDR = 1
**Motoric Frailty**				
58	Europe (SPRINTT Project), 2018	SPPB (score between 9 and 3) and ability to walk for 400 m in <15 min		Occurrence of motoric disability: inability to walk for 400 m in <15 min and/or loss of one or more points of SPPB score
**Cognitive and Motoric Frailty**				
10	Australia (TASCOG), 2005	Instrumented walkway (GAITRite)	GDS	Clinical diagnosis of dementia
60	Canada, 2007	Instrumented walkway (GAITRite)	Self-report cognitive questionnaire	DSM-IV diagnosis of dementia
61	Canada, 2018	Instrumented walkway (GAITRite)	Mini-mental state examination and the montreal cognitive assessment	Onset of motoric cognitive risk syndrome
42	China (SAGE), 2007	4-m timed walk	Self-report cognitive questionnaire	Clinical diagnosis of dementia
86	France (GAIT), 2009	Instrumented walkway (GAITRite)	Self-report cognitive questionnaire	DSM-IV diagnosis of dementia
42	Ghana (SAGE), 2007	4-m timed walk	Self-report cognitive questionnaire	Clinical diagnosis of dementia
88	India (KES), 2011	10-ft timed walk	GDS	DSM-IV diagnosis of dementia
42	India (SAGE), 2007	4-m timed walk	Self-report cognitive questionnaire	Clinical diagnosis of dementia
38	Israel (2 cohorts), 2003	10-m timed walk	GDS	DSM-IV diagnosis of dementia
		Instrumented walkway (GAITRite)	GDS	DSM-IV diagnosis of dementia
27	Italy (InCHIANTI), 1998	4-m timed walk	WHO Disability Scale	DSM-IV diagnosis of dementia
59	Japan, 2008	6-m timed walk	Self-report cognitive questionnaire	DSM-IV diagnosis of dementia
41	Korea (KLOSHA), 2005	4-m timed walk	Self-report cognitive questionnaire	DSM-IV diagnosis of dementia
42	Mexico (SAGE), 2007	4-m timed walk	Self-report cognitive questionnaire	Clinical diagnosis of dementia
42	Russia (SAGE), 2007	4-m timed walk	Self-report cognitive questionnaire	Clinical diagnosis of dementia
42	South Africa (SAGE), 2007	4-m timed walk	Self-report cognitive questionnaire	Clinical diagnosis of dementia
35	Sweden (SNAC-K), 2020	4-m timed walk	Free recall, trail making test part B, category and letter fluency, mental rotation, digit cancelation, and pattern comparison	Diagnosis performed if the score is 1.5 standard deviation below age-specific means on ≥ 1 cognitive domains
6	Switzerland, 2007	Instrumented walkway (GAITRite)	GDS	DSM-IV diagnosis of dementia
51	United Kingdom, 2007	Instrumented walkway (GAITRite)	GDS	DSM-IV diagnosis of dementia
39	USA (CCMA), 2011	Instrumented walkway (GAITRite)	GDS and AD8	DSM-IV diagnosis of dementia

**FIGURE 1:**
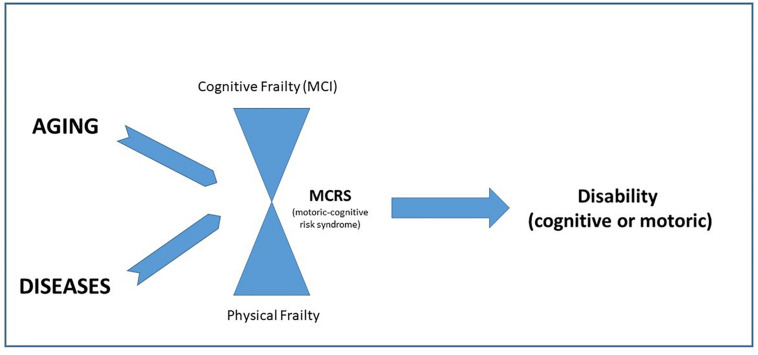
The interaction between cognitive and physical frailty in the evolution toward disability.

Figure 1 underlines the need of a comprehensive evaluation in older persons and the parallel detection of physical and cognitive frailty.

On the one side, low muscle strength could represent the *primum movens* of physical frailty given its role as determinant of slow gait speed, mobility decline, and increased risk of death (Lauretani et al., 2017).

Therefore, hand-grip strength and gait under dual tasking are measurements that should be part of global assessment of MCR syndrome, given their sensitivity to changes in brain function during early stages of the cognitive decline.

From the other side, Osawa et al. (2020) recently published the first longitudinal study in older people testing the correlation between brain volume modifications and changes in muscular strength. These authors found that areas of regional atrophy are related to knee extension isokinetic strength decline, supporting the potential contribution of regional brain atrophy in affecting age-related changes in muscle strength. These results could also imply that a greater rate of strength decline might indicate accelerated shrinkage in brain regions related to motor control (Osawa et al., 2020).

By considering together these findings and bearing in mind that preventing disability is the first goal of geriatric medicine, we should rapidly change our current approach in non-hospitalized patients with comprehensive evaluations and tailored pathways.

There are several European studies focusing on the identification and treatment of the frailty of the older adults and based on an integrated model of care.

In particular, the SUNFRAIL study developed a model and a tool to improve prevention, detection, and treatment of frailty and the management of multimorbidity (Maggio et al., 2020).

### Cross-Talk Between Brain and Skeletal Muscle: The Unifying Role of Exercise and Growth-Neurotrophic Factors

**FIGURE 2:**
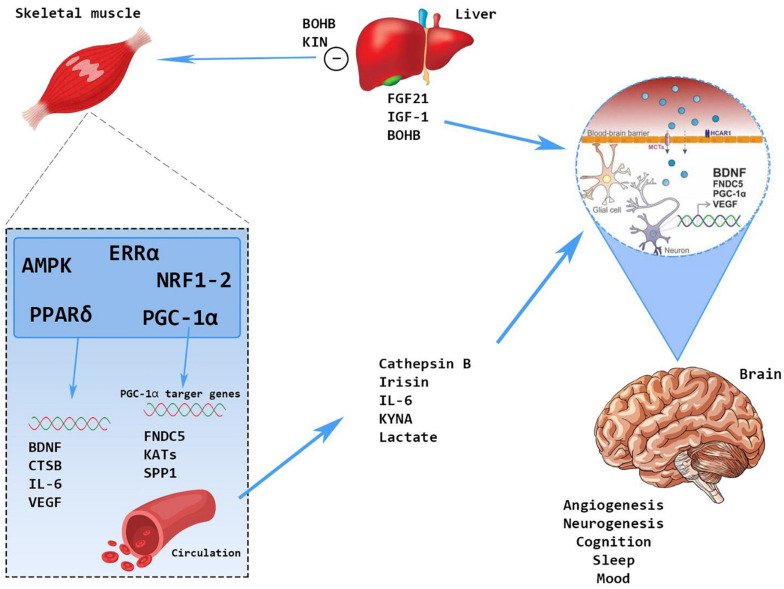
The cross-talk between skeletal muscle and brain: molecular mechanisms.

Physical inactivity and sedentary behavior are among the most important risk factors for disability and dementia. Several chronic diseases such as type 2 diabetes and hypertension accelerate the onset and progression of motoric disability and cognitive impairment. All this information implies that physical exercise can exert a protective action against muscle loss and dementia acting on modulation of endothelial function and cross-talk molecules of the so-called “brain–muscle axis” (Yan et al., 2020) (Figure 2).

Physical activity stimulates brain-derived neurotrophic factor (BDNF) either at central and peripheral level (Delezie and Handschin, 2018).

Central BDNF can use TrkB and p75NRT receptors to improve learning and memory (Yang et al., 2009). Muscle BDNF is produced and secreted by human skeletal muscle in response to exercise. It enhances fat oxidation within the muscle and development of the muscle itself. Moreover, physical exercise directly or indirectly via molecular messengers (the PGC-1 alfa or the AMPK) induce the production of several proteins such as irisin, cathepsin-B, Kina, and β-hydroxybutyrate, all triggers of BDNF production (Boström et al., 2012; Chavan et al., 2016; Moon et al., 2016). The lactates produced in response to physical exercise enhance the production of vascular endothelial growth factor (VEGF) that, together with BDNF and insulin-like growth factor-1 (IGF-1), can increase cell growth and neuronal plasticity (Bibel and Barde, 2000).

### Type of Exercise

Some studies showed that several weeks of resistance exercise in community older persons improve gait and decrease the fall risk (Cadore et al., 2013). In institutionalized older adults with dementia and cognitive impairment, multicomponent exercise has been shown to increase functional capacity and executive functions by decreasing the risk of falling also (Cadore et al., 2014).

Therefore, in patients with dementia, multicomponent exercise program is able to parallel improve cognitive and functional status (Casas-Herrero et al., 2019).

### Future Prospective

Severe forms of global inability are usually triggered by the development of mobility disability. Thus, preventing mobility disability is an important target to prevent advanced disability.

For this reason, a project consisting in a randomized controlled trial (RCT) and named SPRINTT tested the effectiveness of a multicomponent intervention (MCI) in older persons with physical frailty and sarcopenia (Landi et al., 2017).

## Discussion

### Organizational Response Path and Professionals Involved: The Response of Parma Health Trust

All presented data show that the more delayed are the interception and treatment of frailty, the lower are the therapeutic margin and the probability of preventing the commonest frailty adverse events (disability, dementia, and hospitalization).

However, today, the health response to frailty is mainly reactive and is targeting acute late events of frailty. This obviously represents an episodic service completely unable to meet the care needs of these citizens.

A paradigm shift is needed to address the phenomenon of frailty, moving from a “reactive” to a “proactive” model.

Prevention programs and early intervention strategies devoted to face frailty should be implemented in primary care in order to increase the therapeutic margin of these patients and even the appropriateness of hospital admissions (Di Bari et al., 2014).

Nowadays, there are only fragmented care pathways for frailty in the primary care setting, and the existing organizing models consider physical and cognitive domains separately (with Frailty and Motoric Lab devoted to mobility limitation and disability and Cognitive Lab for the rapid assessment and care of Dementia) (Lauretani et al., 2017). These approaches also divide primary care physicians and specialists in Internal and Geriatric Medicine and do not account for the crucial close interaction between domains and disciplines (Lauretani et al., 2017; Grande et al., 2019).

In this perspective, proactive and cost-effective screening programs of both cognitive and physical frailty in older persons would allow the early detection of those who need measures of disability prevention.

For these reasons, the Parma Health Trust of Emilia Romagna Region aims to implement an organizational path that considers both cognitive and physical frailty as a whole, where the community and hospital should fully cooperate in all phases of detection and treatment of frailty by integrating competences and adopting easy to use approaches and methodology.

#### First Phase: Identification of Frailty

The first phase can only start in the context of primary care, the closest context to living environment of the older persons.

Recent studies have shown the efficacy of the SUNFRAIL Screening Tool in appropriately detecting the citizens needing a more in-depth evaluation, thanks to its negative predictive value of 84.6% (Maggio et al., 2020).

This assessment of frailty in the primary care should be conducted by the general practitioner or, when present, a community nurse (Obbia et al., 2020). After the administration of the SUNFRAIL Tool, the suspect of frailty condition should induce these professionals to move into a second-level comprehensive geriatric assessment (Cesari et al., 2016).

This type of assessment, which can be carried out both in the community and in the hospital, should be performed by a multidisciplinary team in order to ensure a combined and in-depth evaluation of motoric and cognitive functions (Pilotto et al., 2017).

During the visit, the following domains should be assessed:

• Physical function: conducted mainly by the geriatrician and the nurse, this type of evaluation must at least investigate the balance, the strength of the lower and upper limbs, and the characteristics of the gait. Crucial is also the pharmacological recognition and reconciliation operated by the geriatrician. This professional figure, when suspects sarcopenia, could prescribe BIA or DEXA examination to confirm the presence of low muscle mass.
• Cognitive function: the neuropsychologist and, where present, the neurologist should perform a complete cognitive, depression, and IADL assessment in order to identify mild cognitive impairment. Brain CT should be included in the diagnostic process together with the assessment of quality of life.

#### Second Phase: Treatment of Frailty

Similarly to diagnostic evaluation, the treatment of frailty requires a multidisciplinary approach, starting from the community that is the ideal setting in this regard.

Physical activity is the most effective treatment for physical frailty. Regular adherence to physical activity programs improves balance, functional autonomy, mood, and cognitive performance (Landi et al., 2017; Alhambra-Borrás et al., 2019; Casas-Herrero et al., 2019).

Depending on the conditions of the patient, physical activity can be administered by motor scientists or physiotherapists, with the potential advice of a physiatrist, and requires the supervision of geriatricians and multiprofessional team for ensuring the safety and the effectiveness of the intervention.

In cases of sarcopenia, physical activity needs to be accompanied by nutritional intervention, held by a nutritionist or a dietician, in order to guarantee the correct intake of protein, essential amino acids, vitamin D, and micronutrients (Landi et al., 2017).

Finally, an intervention conducted in patients at risk of dementia should be based on memory training and managed by a neuropsychologist. Also in these cases, nutritional intervention produces a good response in terms of cognitive performance (Ng et al., 2015).

Three different case scenarios can better explain why the current approach considering physical and cognitive domain separately should be changed in the next future.

### Case Scenarios and Current and Hypothetical Organization Models and Contexts

#### Outpatient Evaluation in the Context of a Clinical Study on Physical Frailty

Male patient, in the age range of 70–80 years old, independent in daily activities with history of falls. This patient was admitted to the Frailty and Morbidity Laboratory of the University-Hospital of Parma, where clinical evaluation was performed (Table 2).

**TABLE 2:** Most relevant parameters of the clinical evaluation performed in Frailty and Multimorbidity Laboratory of Hospital of Parma—first case.

Vital Signs	Blood pressure: 140/90 mmHg; heart rate: 73 bpm; ambient air oxygen saturation: 98%
Physical examination	Normal
BMI	41.54—class 3 obesity
Pharmacological therapy	Atenolol, Doxazosin, Simvastatin
MMSE	30/30
SPPB score	Balance: 4/4; gait speed: 4/4; Chair test: 1/4; Total: 9/12 expressive of physical frailty
ADL	6/6
IADL	8/8
MNA-SF	14/14—no risk of malnutrition

*BMI, body mass index; MMSE, mini-mental state examination; SPPB, short physical performance battery; ADL, activities of daily living; IADL, instrumental activities of daily living; MNA-SF, mini nutritional assessment short form.*

The multidisciplinary team was composed of a geriatrician, a nurse, and nutritionist; the routine biochemical tests were normal. The patient also underwent DEXA scan that was suggestive of sarcopenia according to the Foundation for the National Institutes of Health (FNIH) criteria. The team agreed on the diagnosis of sarcopenia and physical frailty with lower limbs strength as potential factor explaining the history of falls.

A cardiologist visited the patient in order to evaluate the safety of a physical-exercise-based intervention. The ambulatory blood pressure monitoring (ABPM) showed normal blood pressure (126/75 mmHg). The cardiologist also diagnosed a left ventricular hypertrophy not precluding the physical activity intervention.

The patient also underwent a complete nutritional visit in order to adhere to a personalized diet based on caloric restriction but with and adequate protein intake.

Finally, a motor scientist prepared a specific exercise program, composed of aerobic and resistance exercises, with sessions regularly performed in the clinic’s gym and at home.

#### Outpatient Evaluation in a Cognitive Frailty Clinic: Diagnosis and Treatment

Female patient, in the age range of 80–90 years old, living alone, and independent in daily activities.

The patient had history of falls and fractures in the previous 2 years, subjective cognitive decline in the focusing and capacity.

This patient was admitted to the Cognitive Frailty Clinic Hospital of Parma, where a clinical evaluation was performed (Table 3).

**TABLE 3:** Most relevant parameters of the clinical evaluation—second case.

Vital Signs	Blood pressure: 145/80 mmHg; heart rate: 60; oxygen saturation: 97%
Neurological examination	Romberg+
Pharmacological therapy	Folic acid, Propranolol, Lansoprazole, Atorvastatin, Timolol, Mesalazine, Rifaximin, Levothyroxine sodium
MMSE	28/30–27.1/30 adjusted, suggestive of normal cognitive functions
CDT	1/3, suggestive of a cognitive impairment

*MMSE, mini-mental state examination; CDT, clock drawing test.*

The patient underwent second-level neuropsychological assessment, which revealed the presence of multiple cognitive deficits (linguistic, praxic, attentional, and executive) and, together with preserved functionality, allowed the suspicion of extra-mnestic MCI, minor neurocognitive damage.

The multidisciplinary team, composed of a geriatrician, a nurse, and a neuropsychologist, agreed to suggest a cognitive stimulation-training-based intervention and a close follow-up as also suggested by the Interceptor project having Parma as participating sites (Rossini et al., 2019).

#### Integrated evaluation of Physical and Cognitive Frailty: A Future Model

Female patient, in the age–range of 80–90 years old, independent in daily activities.

The patient has history of falls and fractures in the previous 2 years, subjective cognitive decline in the mnestic domain.

In this patient, a first clinical evaluation was performed in the context of primary care setting. Then, she was admitted to the Frailty and Morbidity Laboratory of Hospital of Parma, with blood chemistry evaluation (Table 4).

**TABLE 4:** Most relevant parameters of the clinical and biochemical evaluation—third case.

Vital Signs	Blood pressure: 150/80 mmHg; heart rate, 70; ambient air oxygen saturation: 97%
Physical examination	Normal
Biochemistry analysis	Total cholesterol, 203 mg/dl; triglycerides, 168 mg/dl; Mg^2+^, 3.5 mg/dl; vitamin D, 23 ng/ml
Pharmacological therapy	Alendronate and cholecalciferol (for 10 years), SSRI, benzodiazepine as needed, statin and acetylsalicylic acid
MOCA	16.5, suggesting a scarce performance in visual–spatial, mnestic and temporal orientation domains.
SPPB score	Balance: 4/4; gait speed: 4/4; chair test: 3/4; total: 11/12 expressive of absence of physical frailty
ADL	6/6
IADL	6/8

*SSRI, selective serotonin reuptake inhibitors; BP, blood pressure; HR, heart rate; SPPB, short physical performance battery; Mg, magnesium; MOCA, Montreal Cognitive Assessment; ADL, activities of daily living; IADL, instrumental activities of daily living.*

Alendronate was deprescribed given the 7-year treatment in the history and low vitamin D levels, and the patient underwent second-step analysis as reported in Table 5.

**TABLE 5:** Second-level cognitive–physical assessment.

**Brain CT scan**	**No signs of cerebrovascular disease.**
Nutritional status assessment and anthropometry	BMI, 25.6; MNA-SF: 13/14; analysis of 3-day dietary records revealed a total kcal/day: 1,340 (25–30 kcal/kg with a daily protein intake was 0.88 g/kg body weight).
Body composition and sarcopenia assessment	SMI, 6.78 kg/m^2^ obtained by BIA; handgrip test, 11 kg
Neuropsychological evaluation	Multiple cognitive impairments mnestic and extramnestic (executive and praxic), with a reduction in the instrumental activities of daily living
NPI	26/144, moderate anxiety, disinhibition, irritability associated with moderate aberrant motor activity

*CT, computed tomography; MNA, mini nutritional assessment; SMI, skeletal mass index; BIA, bioelectrical impedance; NPI, neuropsychiatric inventory.*

The multidisciplinary team was composed of a geriatrician, a neuropsychologist, a nutritionist, and a physical therapist. The multidisciplinary team agreed on the diagnosis of major cognitive disorder associated with behavioral and psychological symptoms of dementia and muscle dysfunction with lower handgrip strength. This pathological condition could have explained the history of falls and allowed the diagnosis of possible/probable Alzheimer’s disease.

The correct treatment of major neurocognitive disorder (pharmacological and non-pharmacological) and psychological symptoms of dementia was started by the multidisciplinary team.

The pharmacological therapy can be summarized as follows:

(1) Evaluation of the current pharmacological treatment with deprescription of alendronate and beginning of 25-hydroxycholecalciferol vitamin D supplementation to reduce the risk of osteoporotic fractures and falls;
(2) Specific treatment with acetylcholinesterase inhibitors, after a cardiac examination.

Non-pharmacological intervention consisted of motoric exercises for improving balance, motor coordination, and ability on ideation of motoric programs.

## Conclusive Remarks

This paper aims to show a possible treatment model based on integrated motoric cognitive approach in order to stimulate this new vision and to proactively manage community-dwelling older persons with suspected frailty.

## Author Contributions

FL and MM contributed to the conception and design of the work, the acquisition, analysis, and interpretation of data for the work, drafting the work or revising the work critically for important intellectual content, final approval of the version to be published, and agreement to be accountable for all aspects of the work in ensuring that questions related to the accuracy and integrity of any part of the work are appropriately investigated and resolved. YL, FFP, AD, CF, RF, EG, UL, GR, MR, MS, IZ, and GP contributed to the acquisition, analysis of data for the work, revising the work critically for important intellectual content, and final approval of the version to be published. All the authors have read and agreed to the published version of the manuscript.

## Conflict of Interest

The authors declare that the research was conducted in the absence of any commercial or financial relationships that could be construed as a potential conflict of interest.
